# Rare Earth Element-Induced Condensation of the Block V of the Repeats-in-Toxin Domain from CyaA from *Bordetella pertussis* for Separations

**DOI:** 10.1021/acs.langmuir.5c04148

**Published:** 2025-12-18

**Authors:** Luis E. Ortuno Macias, Farid Khoury, Mrinal K. Bera, Wei Bu, Binhua Lin, Scott Banta, Raymond S. Tu

**Affiliations:** a Department of Chemical Engineering, 14770 The City College of New York, New York, New York 10031, United States; b Department of Chemical Engineering, 5798 Columbia University, New York, New York 10027, United States; c NSF’s ChemMatCARS, Pritzer School of Molecular Engineering, 2462 University of Chicago, Chicago, Illinois 60637, United States

## Abstract

Rare earth elements (REEs) are critical for the development of a range of new technologies. However, the current industrial separation processes of these metals from natural sources, recycled materials, and industrial effluents involve the large consumption of organic solvents, resulting in a sizable environmental footprint. We aim to exploit the high affinity of the block V peptide of the repeats-in-toxin (RTX) domain of the adenylate cyclase protein from *Bordetella pertussis* for the separation of REEs. This peptide selectively binds with lanthanide (Ln) cations and can undergo Ln-induced phase separation, which can be used in bioseparation processes. Here, we evaluated the self-assembling structures of complexes of the RTX domain peptide folded in the presence of Ln^3+^ cations. Size distribution and surface potential measurements of complexes were taken to understand the Ln-induced changes in the complexed peptide. Transmission electron microscopy imaging was used to explore the structures of complexes, while anomalous small-angle X-ray scattering measurements were used to determine the distribution of Ln^3+^ ions within the protein-based macrostructures. In the presence of excess Ln^3+^, we observed the formation of coral-like cylindrical structures comprised of Ln^3+^-RTX complexes, with approximately eight trivalent metals per peptide within the nanosized assemblies. These findings provide new insights into the structural organization of assembled RTX domains and their ability to coordinate with REEs, forming nanosized, metal-rich structures that naturally condense, providing a proof-of-concept for protein-based separation processes of these critical materials.

## Graphical abstract

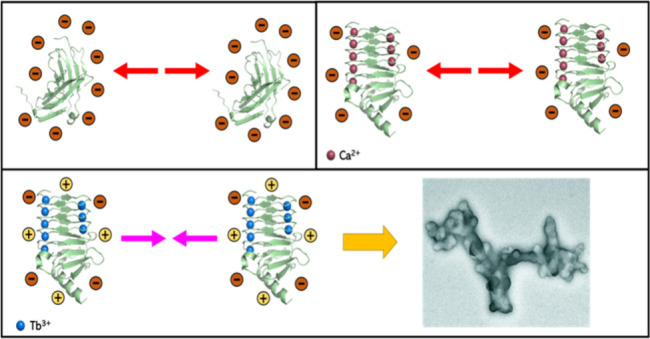

## Introduction

Rare earth elements (REEs) are those between lanthanum and lutetium on the periodic table, also known as lanthanides.^1^ Yttrium and scandium are included in this category of elements as they share chemical and physical similarities with the group and exist in nature with the lanthanides (Ln^3+^).^2^ The unique properties of REEs make their use possible in a wide range of industrial applications, such as electronics, catalysis, clean energy, batteries, and magnetics.^1–4^ The technologies and mineral processing implemented for obtaining concentrated solutions of REEs depend on the type of ore or mineral being processed. Ore beneficiation, mineral concentrate decomposition, and rare earth leaching are techniques that are commonly used for the preconcentration of REE solutions.^5,6^ Likewise, several chemical separation techniques can be used for the separation of REEs from concentrated solutions, such as ion exchange, chromatography, and solvent extraction processes.^7^ The solvent extraction separation process takes advantage of the ability of metal ions to be transported across the interface between an aqueous solution and a nonmiscible organic solution. Generally, the aqueous solution contains REE ions and soluble impurities, while the organic solution contains an extractant. The desired solute (REE ions) is initially dissolved in the aqueous solution, but eventually is distributed between the two phases until equilibrium is achieved.^8^

Separation and purification of REEs have relied upon solvent extraction because of the advantages of the process, such as simple, fast, continuous operation, mild process conditions, and inexpensive handling of large quantities of materials.^9,10^ Solvent extraction processes for the separation of REEs require a large volume of solvents due to the high viscosity of the extractants used for the collection of the desired metals. Currently, a key environmental impact is the solvent extraction stage.^11^ Solvent extraction in the separation and purification of REEs from bastnaesite significantly impacts the environment, contributing approximately 75% to the terrestrial acidification, over 60% to the global warming potential, more than 50% to terrestrial, freshwater, and marine eutrophication, and around 70% to water resource depletion. These impacts are relative to the entire REE extraction process, which includes mining, beneficiation, acid roasting, leaching, and solvent extraction.^12^ However, solvent extraction in the separation and purification of REEs significantly impacts the environment, contributing to acidification, global warming potential, and eutrophication.^12^ These challenges have encouraged the search for alternative, more sustainable methods of REE coordination and recovery.

Over the past two decades, the characterization of the lanthanome, proteins involved in the recognition, uptake, and usage of lanthanides, has become a wider field of research. In addition to the discovery, engineering, and use of these new biomolecules, scientists have taken advantage of the chemical similarity of lanthanides to calcium cations and investigated Ln^3+^ coordination within a number of calcium-binding proteins. Some metalloproteins, such as calmodulin, parvalbumin, calcineurin, calbindin, S100β, troponin C, and cadherins, have been shown to bind with Ln^3+^ cations similarly to Ca^2+^, generally with a stronger affinity due to the greater electropositivity of Ln^3+^ over Ca^2+^.^13–19^ The high selectivity of REE-binding peptides and proteins has encouraged the exploration of both designed and naturally occurring molecules for the selective separation of REEs.^20–26^ However, many of these separation processes require the immobilization of bioextractants onto synthetic and biological materials. This immobilization can influence the structure and function of these molecules, potentially altering their stability, conformation, or activity. Studies have shown that immobilized proteins may retain their conformation but exhibit changes in orientation, as observed for the B1 domain of protein G, or experience activity variations depending on immobilization conditions, as seen with β-galactosidase.^27^ Immobilization of the lanthanide-binding protein Lanmodulin (LanM) via thiol-maleimide click chemistry has led to the destabilization or inaccessibility of one metal-binding site, resulting in the binding of only two out of the three metal ions that are typically bound by the free peptide in solution.^28^ Immobilization of LanM proteins using SpyTag-functionalized magnetic nanoparticles and LanM-SpyCatcher achieved 80% of the adsorption activity of free LanM-SpyCatcher, with binding loss attributed to steric hindrance affecting the metal-binding pockets.^29^ Beyond full-length proteins, smaller lanthanide binding tag (LBT) peptides derived from EF-hand motifs^30,31^ have been explored for their ability to coordinate REEs. These short peptides are of particular interest for separation technologies due to their ease of synthesis, modularity, and ability to maintain lanthanide affinity while reducing complexity.^32^ LBTs have been expressed on cell surfaces to enhance bioadsorption of REEs.^33–36^ However, quantitative assessments of how immobilization on biological surfaces affects the LBT binding affinity remain limited. While adsorption capacity is often improved through cell surface display, steric hindrance, local charge effects, and peptide conformational changes may contribute to variations in affinity that have yet to be fully characterized.

In contrast, investigations into the peptide binding loop of the EF hand of LanM (16 amino acids) tethered to solid surfaces have provided clearer insights into binding behavior of short peptides upon immobilization.^37^ QCM-D analysis confirmed that surface immobilization did not significantly alter the coordination properties. This retention of affinity may result from peptide linker flexibility, optimized attachment orientation, and the ability to adopt a binding-competent conformation. Taken together, these studies highlight the importance of how immobilization influences the performance of REE-binding biomolecules.

The block V of the repeats-in-toxins (RTX) domain of the adenylate cyclase (CyaA) protein from *Bordetella pertussis* folds into a β-roll secondary structure upon Ca^2+^ binding^38–40^ (Figure 1). Previous studies using inductively coupled plasma optical emission spectroscopy (ICP-OES), Förster resonance energy transfer (FRET) efficiency, and circular dichroism (CD) spectroscopy have demonstrated that this peptide binds lanthanide cations, with a binding capacity similar to that of calcium at a pH of 5.5 but with higher affinity than Ca^2+^ and other non-REE trivalent and tetravalent metals. Also, the RTX domain folds upon Ln^3+^-binding into a stable secondary structure that differs from the Ca^2+^-peptide conformation and with a more ordered structure.^41^ In addition to its high affinity for lanthanides (reflected in the parabolic trend of apparent dissociation constants (*K*_D_) across the lanthanoid series as measured by FRET), the recombinant expression of the RTX peptide offers significant practical advantages. These include cost efficiency, scalability, and a lower environmental impact compared with synthetic molecule production. Importantly, prior characterizations have also shown that the RTX peptide exhibits significantly lower binding affinity for other high-valency metal ions such as Al^3+^, In^3+^, and Th^4+^.^41^ This selectivity highlights its utility as a model system for understanding lanthanide-peptide coordination.

**Figure 1:**
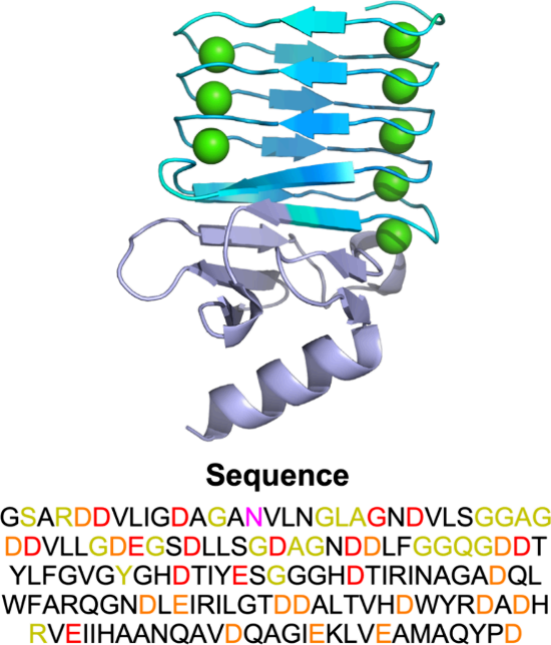
X-ray crystallographic structure of block V of the RTX domain of CyaA complexed with Ca^2+^ cations (PDB: 5CVW). Calcium ions are colored green, β-roll forming amino acids blue, and capping group residues in purple. Amino acid sequence of the peptide is presented below the coordinated structure in the figure. Negatively charged amino acids with COO^–^ side chain groups coordinating with Ca^2+^ cations are colored red, amino acids with CO backbone groups coordinating with Ca^2+^ cations are colored yellow, amino acids with CO side chain groups coordinating with Ca^2+^ cations are colored pink, and negatively charged groups not coordinating with Ca^2+^ cations are colored orange.

RTX repeats can also self-associate upon metal binding, forming aggregates and condensed phases in solution. While aggregation is often associated with structural instability, certain peptides retain well-defined secondary structures in aggregated states, as observed for amyloid β-fibrils.^42,43^ Given that RTX repeats adopt ordered structures upon lanthanide binding,^41^ it is reasonable to hypothesize that metal-induced aggregation may preserve the integrity of the binding sites while organizing the peptides into higher-order assemblies.

In this study, we exploit the high affinity of block V of the RTX domain of CyaA for Ln^3+^ cations and promote the metal-directed self-association of individual complexes into assemblies that result in a macrophase separation, and hence a spontaneous condensation of structures rich in Ln^3+^ cations. While this work serves as a foundational scaffold for developing improved strategies for the separation of REEs, with further characterization needed to refine and optimize this approach, it also breaks new ground by focusing on the structural and compositional details of Ln-driven RTX assemblies formed in solution. To this end, we characterize the resulting soluble nanostructures using dynamic light scattering (DLS) to identify structural and surface charge changes induced by multivalent metals. Additionally, we image the metal-directed condensates resulting from concentrated solutions of peptide and Ln^3+^ by transmission electron microscopy (TEM) and analyze the spatial distribution of Ln^3+^ using anomalous small-angle X-ray scattering (ASAXS). These findings bridge a critical knowledge gap, advancing our understanding of how metal-driven peptide assemblies behave in solution and how this behavior can be controlled and optimized for REE separation applications.

## Results and Discussion

### Self-Association and Surface Charge Neutralization of RTX Peptide upon Binding with Lanthanides

The RTX peptide is largely disordered in the absence of multivalent cations. This peptide is negatively charged at pH 5.6, which prevents secondary structure formation due to intermolecular electrostatic repulsions. The charged residues should counteract aggregation by the repulsive effect of their charges and contribute significantly to the solubility of the peptide. The hydrodynamic diameters of RTX peptide at a concentration of 1 μM at three conditions, (1) without any multivalent cations, (2) in the presence of Ca^2+^, and (3) in the presence of Tb^3+^ ions, are shown in Figure 2A. In the absence of multivalent cations, dynamic light scattering indicates that the peptide is in an oligomeric state and is not as stable as monomers, with an intensity peak indicating structures with a hydrodynamic diameter of 451 ± 48 nm (shown on the column with the gray color background, Figure 2A). This oligomeric state is inferred from the fact that the peptide, composed of approximately 200 amino acids, would have an estimated maximum length of only about 72 nm if fully extended (assuming approximately 0.36 nm per residue), which is substantially smaller than the observed DLS value. The self-association observed for the unbound peptide is a well-documented characteristic of the full-length CyaA protein when refolded by dialysis, dilution, or buffer exchange,^44,45^ and it has been reported for other RTX peptides in the apo-state as well.^46,47^ Such a behavior has been attributed to the presence of localized β-structures populations within these domains^47^ as well as the high content of solvent-exposed aromatic residues.^48^ Figure 2A (column with the orange color background) shows that the molecule forms oligomeric structures in the presence of Ca^2+^ cations at concentrations sufficient to saturate the peptide based on the measured affinity constant, *K*_D_ = 460 μM.^41^ The oligomeric structures observed for Ca^2+^ binding have a hydrodynamic diameter of 455 ± 49 nm. Dimension similarities between cation-free and the Ca^2+^-bound aggregates indicate that the peptide’s structural changes induced by divalent metal-association do not affect the size of the self-associated structures. Previous work had demonstrated that the RTX peptide can bind with lanthanide cations similarly to the divalent cation Ca^2+^, but with higher affinities toward the lanthanide metals.^41^ Hydrodynamic diameters of structures formed by the RTX domain upon Tb^3+^ binding are shown in Figure 2A (column with the purple colored background). In contrast to the calcium-bound structures, a monomodal distribution is observed (see Figure S1) with a peak corresponding to larger aggregates (hydrodynamic diameter of 1195 ± 129 nm) compared to the cation-free and the Ca^2+^-coordinated peptides. The correlation functions corresponding to the DLS measurements are shown in Figure S2. The micrometer-sized structures in the presence of Tb^3^^+^ cations may arise from the trivalent nature of Tb^3+^ compared to divalent ions like Ca^2+^. The higher charge density of Tb^3+^ allows it to bind to more oxygen atoms from the negatively charged carboxylate groups (aspartic acid and glutamic acid residues not coordinating with Ca^2+^, shown in orange in the sequence presented in Figure 1), effectively neutralizing the complex. This behavior is consistent with well-established mechanisms describing multivalent ion-driven condensation and aggregation of polyelectrolytes, where counterion correlations and charge neutralization promote intra- and interchain association.^49–52^ The crystal structure of the calcium-bound peptide reveals that two of these amino acids, which do not coordinate with calcium, instead form complexes with Mg^2+^ ions present in the crystallization solution (PDB: 5CVW). This observation strengthens the hypothesis that these noncalcium-bound charged groups can also coordinate with metal ions such as Tb^3+^ in our system.

**Figure 2:**
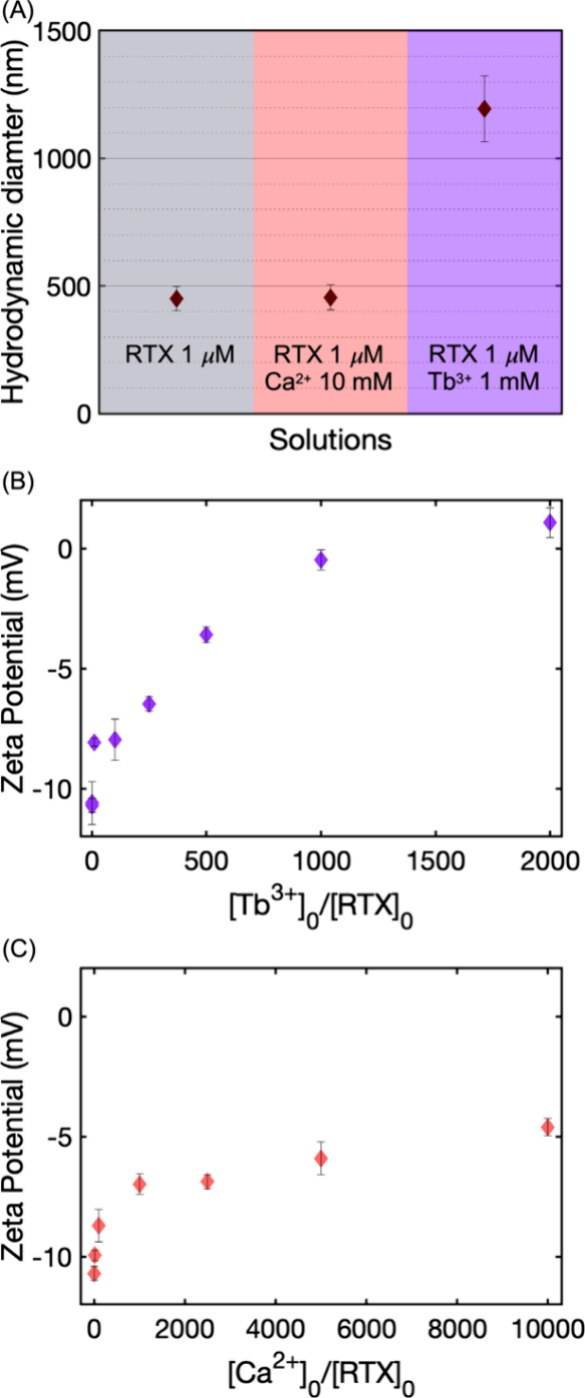
(A) Average hydrodynamic diameter from different solutions containing 1 μM of RTX peptide with no multivalent cations and with Ca^2+^ or Tb^3+^ cations; intensity size distribution for the same solutions are represented in Figure S1. The error bars corresponding to the hydrodynamic diameter are the standard deviation, reported by the equipment as a measure of the spread of dispersion of particle sizes within a sample for multiple records. (B) Zeta potential from solutions containing 1 μM RTX peptide and different concentrations of Tb^3+^, with a maximum concentration of cations enough to saturate the peptide. (C) Zeta potential from solutions containing 1 μM RTX peptide and different concentrations of Ca^2+^, with a maximum concentration of cations enough to saturate the peptide. The error bars corresponding to the zeta potential measurements represent the standard deviation from multiple measurements. All peptide solutions, including those with no added ions and those with varying ion concentrations, were prepared in buffer at pH 5.6, and the pH was confirmed to remain unchanged after ion addition.

To provide direct experimental evidence of the charge effect on the nanometer-to-micrometer transition for RTX domain peptides upon Tb^3+^ binding, the surface charge of unbound and metal-bound peptides was studied. The zeta potential of the RTX domain peptide at a concentration of 1 μM and different concentrations of Tb^3+^, including a maximum concentration of ions that ensures saturation of the peptide (with only 1.14% unbound peptide) based on the measured affinity constant (*K*_D_ = 23 μM for Tb^3+^),^41^ were measured, and the results are presented in Figure 2B. The unbound peptide has a negative zeta potential value, in agreement with the negative net charge of the molecule at a pH of 5.6. The zeta potential increases with increasing Tb^3+^ cations in solution until reaching a value close to 0 mV at 2000 μM of Tb^3+^. Zeta potential values approaching 0 mV indicate that the particles in solution are not stable because of the lack of electric repulsion. Therefore, the Tb^3+^-coordinated RTX peptide can undergo structural changes and intermolecular interactions that yield micron-sized structures. The changes of the zeta potential for the RTX domain peptide upon Ca^2+^ binding are also measured to determine the role of cation valency in charge neutralization. The highest concentration of Ca^2+^ used was 10 mM to ensure saturation of the peptide according to previously measured dissociation constants (*K*_D_ = 460 μM for Ca^2+^).^41^ In agreement with Tb^3+^, Ca^2+^ can induce a reduction in the surface charge of molecules and complexes in solution, resulting in a possible destabilization of species. However, in the Ca^2+^-near-saturated system (Figure 2C), the surface charge of the species in solution remains negative (zeta potential of −4.6 ± 0.4 mV), indicating that the complexes are not fully neutralized due to the lower valency of the Ca^2+^ cations. This contrasts with the Tb^3+^ system, where a more complete neutralization is observed. The enhanced effectiveness of Tb^3+^ in charge screening is attributed to its higher charge and smaller ionic radius, which together result in a higher charge density. This enables stronger and more extensive coordination with negatively charged carboxylate groups on the peptide, facilitating ion-induced aggregation.

### Lanthanide-Induced RTX Domain Peptide Condensation

We have shown that RTX peptides form micrometer-sized structures when bound to Tb^3+^ cations, possibly due to the neutralization of charges of complexes, which gives rise to a boost of intermolecular attractive forces between the nonpolar residues of complexes. While such charge neutralization is evident at high terbium concentrations, the formation of these structures might also be influenced by conformational changes in the RTX peptide upon lanthanide coordination. Circular dichroism studies have shown that lanthanide binding can induce a more ordered secondary structure compared to calcium-bound forms, which could potentially contribute to the observed aggregation behavior.^41^ To further enhance these hydrophobic interactions to form large-scale structures that further condense, which is desired for an efficient separation process, molecular association is promoted on a larger scale by increasing the concentration of the peptide and trivalent cations in solution. Figure 3A shows that no visible aggregates are observed for a solution containing 20 μM RTX domain peptide. At this concentration, nanometer-sized amorphous structures are observed by TEM and shown in Figure S3. With the addition of Tb^3+^ cations into the solution, the formation of visible aggregates (cloudy solution) is observed immediately, with a rapid increase in the density of these structures that condensed further into a turbid liquid macrophase that spontaneously settled to the bottom of the tube without centrifugation (see Figure 3B, 2 h after introduction of Tb^3+^ into the solution). Note that similar condensation behavior was also observed when Lu^3+^ cations were introduced at the same concentration as Tb^3+^ (0.5 mM), whereas no visible aggregation or phase separation occurred in the presence of Ca^2+^ at concentrations of up to 20 mM, indicating that this condensation process is specific to trivalent lanthanide coordination.

**Figure 3:**
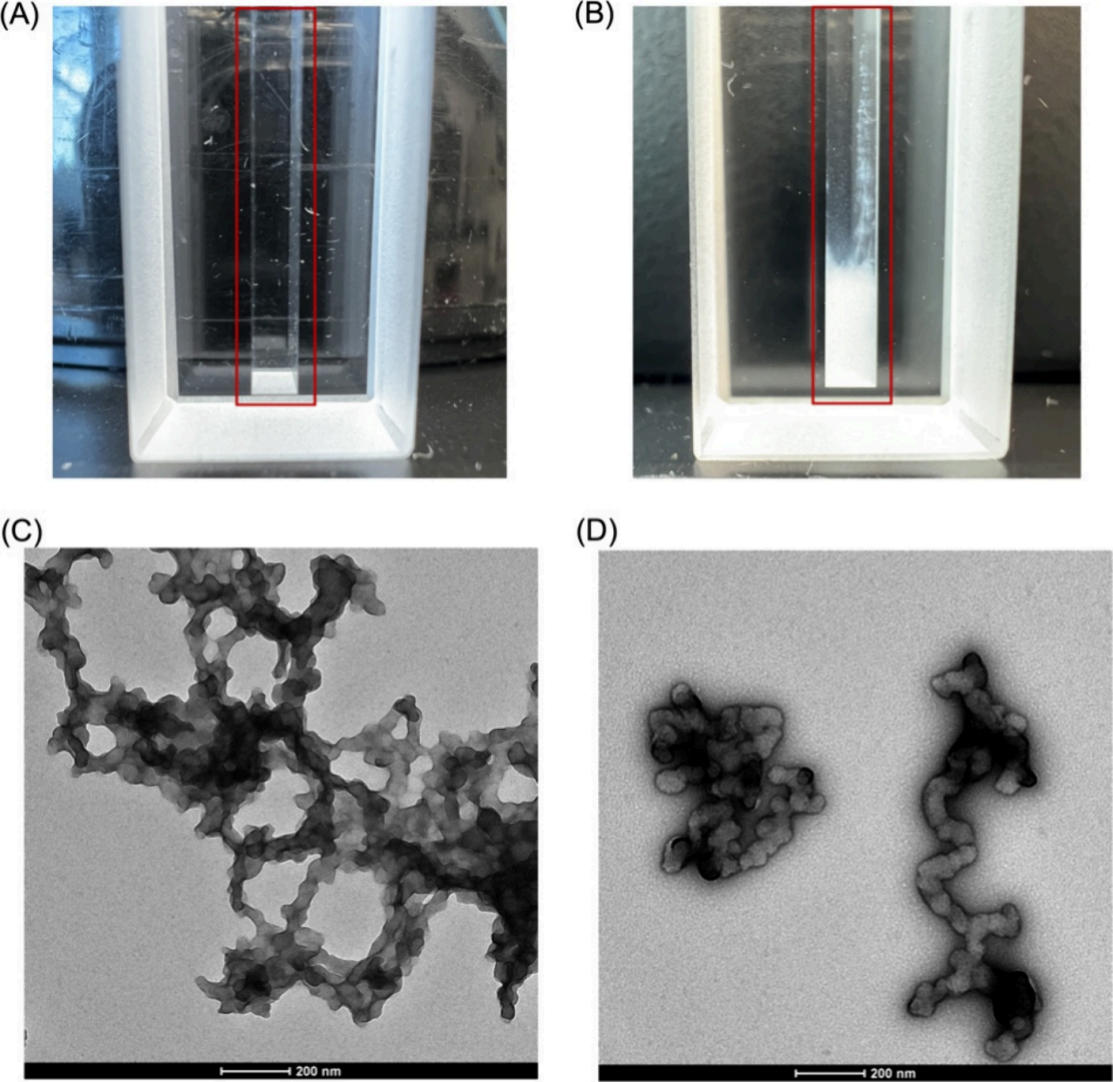
(A) Solution containing 20 μM RTX peptide at pH 5.6. (B) Solution containing 20 μM of RTX peptide and 0.5 mM of Tb^3+^ at pH 5.6, showing the macrophase-separated condensates settled at the bottom of the tube. The solutions were placed in spectroscopy cells for a clear visualization, with red boxes highlighting the sample in each panel. This condensation behavior was reproducibly observed in at least three independently prepared samples under the same conditions (Figure S4). Consistent aggregation and phase separation were also observed in samples prepared for ASAXS and electron microscopy analyses, including both single and mixed lanthanide systems. (C, D) TEM images of dried samples from a solution containing 20 μM of RTX peptide and 0.5 mM of Tb^3+^ at pH 5.6. Both panels (C) and (D) correspond to the same Tb^3+^-containing sample and show different regions of the same TEM grid, highlighting the morphology of the aggregated structures. Similar structures were observed for solutions containing 50 μM of RTX peptide and different [Tb^3+^]_0_/[RTX]_0_ ratios; these results are presented in Figure S5. The scale bar for parts C and (D) represents 200 nm.

TEM shows the morphology of structures resulting from Tb^3+^ binding. Figure 3C shows that the addition of trivalent cations to a concentrated solution of RTX domain peptide causes the condensation of the peptide into polydisperse “coral-like flexible fibrils” with diameters between 50 and 80 nm. The interconnected structures may be the result of the drying process required for sample preparation and subsequent imaging. Several single smaller structures are observed during the sample inspection (see Figure 3D), which supports the premise that the macrostructures observed in Figure 3C are the result of individual aggregates packed together due to their high density in solution and the drying process. TEM imaging from a solution containing 20 μM of the RTX with 20 mM of Ca^2+^ shows structures that are morphologically distinct from those observed in the presence of lanthanides (Figure S6) and instead resemble the appearance of the unstructured peptide at high concentrations (Figure S3), suggesting that the aggregation and morphology observed with lanthanides are specific to REE binding. Cryo-electron microscopy (cryo-EM) of RTX-Ln solutions at concentrations comparable to those used in the dried TEM samples reveals similar networked and fibrillar morphologies (Figure S7), confirming that these structures exist in solution and are not artifacts introduced during sample preparation.

While β-sheet-rich structures tend to form ordered fibrils in supramolecular assemblies,^53^ we do not observe the presence of well-defined fibrils on the imaged aggregates. Notably, the fibrillization propensity and resulting fiber morphology of β-sheet structures can be influenced by conformational changes of monomers induced by the ionic strength of the solution,^54^ binding of metal ions,^55–57^ pH,^58,59^ and temperature.^58^

### Lanthanide Spatial Distribution in Macromolecular Structures

Experimental results show that lanthanide cations are responsible for the aggregation and condensation of RTX domain peptides. Moreover, ASAXS measurements are taken to understand how these metals are distributed within the aggregates and to establish whether lanthanide cations play a direct or indirect role in the formation of supramolecular structures. ASAXS allows the concentration of Ln^3+^ to be determined within the self-assembling structures, as well as the ratio between the electron density of these elements and the electron density of the organic structures. While conventional small-angle X-ray scattering (SAXS) provides structural information averaged over all scattering species in solution, ASAXS extends this technique by exploiting the energy dependence of X-ray scattering near an element’s absorption edge. By measuring the scattering intensity at several photon energies close to this edge, it becomes possible to deconvolute the total signal into distinct contributions corresponding to the resonant element (e.g., Tb^3+^ or Lu^3+^) and the surrounding matrix (e.g., the peptide). This capability allows ASAXS to probe the spatial distribution, density, and association of metal ions within complex macromolecular or colloidal systems, providing insight into how specific elements participate in self-assembly or structural organization.

Figures 4A and B show the ASAXS profiles of the aggregates formed from a solution containing 20 μM RTX domain peptide at pH 5.6 with 0.5 mM TbCl_3_ and 0.5 mM LuCl_3_, respectively. Terbium and lutetium were chosen for the study due to their higher affinity of the peptide with these metals compared to the rest of the lanthanides.^41^ The scattering intensity is divided into three terms: normal SAXS term, resonant term, and cross term. The normal SAXS term accounts for the elastic scattering of X-rays from the electrons in atoms that make up all of the species in the sample. The scattering intensity derived from this term can give information on the size, morphology, and molecular mass of the scattering species.^60^ The resonant term contains information about the spatial distribution of the resonant scattering atoms only, in this case, the Tb^3+^ ions. Finally, the cross term is constituted by the scattering of both the resonant term (Tb^3+^) and the peptide. The shape and slope of the SAXS term data shown in Figure 4, corresponding to the Tb^3+^- and Lu^3+^-induced aggregates, can give information about the morphology of the structures in solution. Based on the TEM structures from Figure 3C,D, either rodlike or cylindrical structures are expected from the shape analysis of the SAXS term curve. However, the *Q* range that determines the morphology of a rod is between *Q* = 1/*L* and *Q* = 1/*D*, where *L* is the length, and *D* is the diameter of the rod,^61^ which complicates the analysis since the length of the structures is not well-defined from the TEM analysis. Nevertheless, the shapes of all SAXS profiles studied in this work are in good agreement with SAXS profiles from fibril structures reported elsewhere.^62,63^ While the dimensions of structures are part of the results from fitting the three terms that constitute the entire ASAXS data, the SAXS term supports the formation of irregular fibril structures from the aggregation of Ln^3+^ cations and RTX peptides. [Fig fig4]

**Figure 4:**
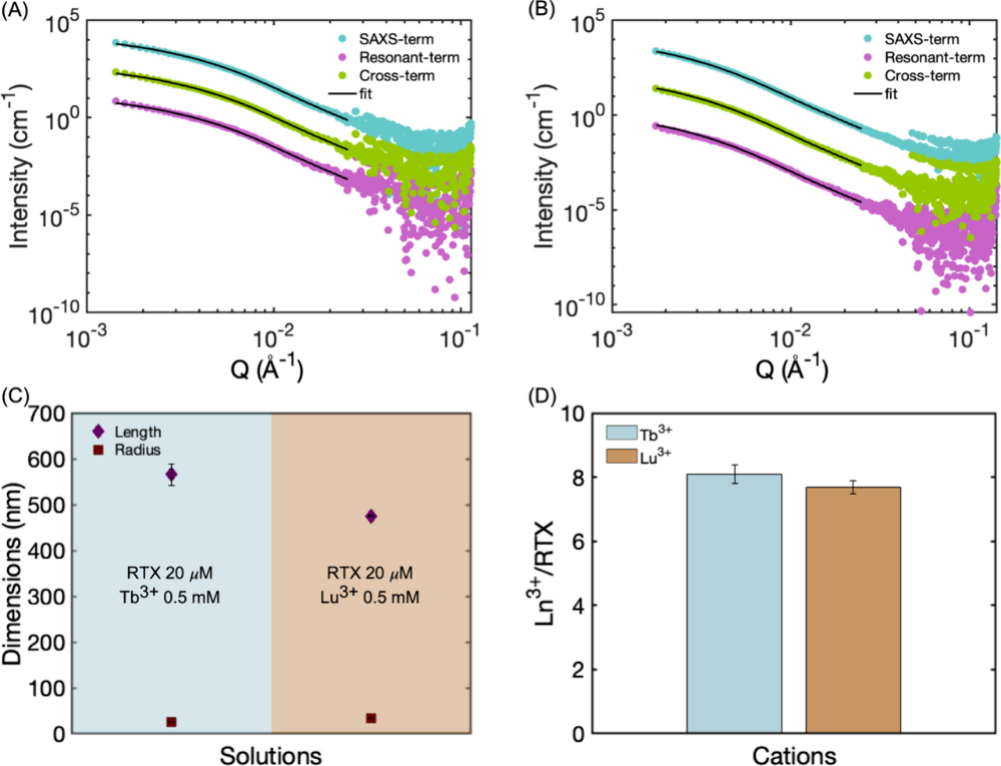
Representative ASAXS profiles and the corresponding fits for a cylindrical model from solutions containing 20 μM of RTX domain peptide at pH 5.6 and (A) 0.5 mM of Tb^3+^ cations and (B) 0.5 mM Lu^3+^ cations. (C) Length and radius of aggregated structures from solutions containing 20 μM of RTX domain peptide at pH 5.6 and either 0.5 mM of Tb^3+^ cations or Lu^3+^ cations, obtained from ASAXS measurements. (D) Ratio Ln^3+^/RTX from solutions containing 20 μM of RTX domain peptide at pH 5.6 and either 0.5 mM of Tb^3+^ cations or Lu^3+^ cations, obtained from ASAXS measurements. Errors in the fitted parameters are obtained by mapping the chi-squared space.

By using a Uniform_Cylinder model available in the XModFit software,^64^ which was selected based on the fibrillar structures obtained from TEM, the scattering distribution profiles were fitted (Figure 4A,B). The details of the model are described in recent work analyzing the double helical structure of silver-modified DNA.^65^ Dimensions of structures, radius, and length of cylinders were obtained for samples containing 20 μM of RTX domain peptide at pH 5.6 and either Tb^3+^ or Lu^3+^ cations at a concentration of 0.5 mM, and are shown in Figure 4C. These parameters were fitted along with the aggregate density, the density of lanthanide cations external to the aggregates, and the peptide-to-lanthanide ratio within the aggregates. During the fitting process, the diameter was initialized close to the average value obtained from TEM measurements (typically within ± 20%), while the length was adjusted to reproduce the overall shape of the scattering curve, particularly the position and slope of the main features. The density and peptide/Ln ratio parameters were refined to account for the intensity differences between the distinct scattering components. To ensure reliable convergence, multiple fits were performed using varied starting values. The fits consistently converged to values within the reported standard deviations. Fits that yielded unphysical results (such as unrealistically high terbium concentration within or outside the aggregates) were discarded, and the fitting was restarted with refined initial conditions until a physically meaningful convergence was achieved.

The mean radii values of 256 ± 5 and 341 ± 1 Å (in agreement with the radial dimension of structures observed from TEM), and the length between 5665 and 4757 Å were found from the morphology dimensions analysis. Morphological similarities between aggregate structures in solutions containing two different cations indicate that the size and shape of the aggregates are not significantly affected by the different trivalent lanthanides coordinating with the peptide but suggest that the morphology of the structure is defined by the conformational changes of the monomer upon binding the trivalent ion. It is important to note that features on the scattering components expected for monodispersed cylindrical structures are absent due to the polydispersity of the structures in solution, in agreement with the TEM images; therefore, a Log-Normal radial size distribution, provided in Figure S8, is used to account for the observed polydispersity. Note that the reported uncertainties in radius appear small; these reflect the confidence in the model fitting and not the actual polydispersity of the system; both samples exhibit broad radial distributions (Figure S8), indicating that the aggregates are similarly heterogeneous in size.

The resonant term, represented in Figure 4A,B, is the scattering contribution from either Tb^3+^ or Lu^3+^ distributions within or around the aggregated structures. The distribution of metals was obtained quantitatively by fitting all the scattering contributions to the Uniform_Cylinder model, where the cations could be within and outside the cylindrical core. Ln^3+^ concentration profiles obtained from the fitting process indicate that cations are uniformly distributed along the core of the cylinder (see Figure S9A). The electron density profile (EDP) of the system, which represents the electrons of all the species in the system per unit volume, is also constructed from fitting the ASAXS data (Figure S9B). The greater electron density observed for the Lu^3+^-bound structures over the Tb^3+^-bound ones is due to the larger effective electron density of the heavier metal Lu^3+^. As for the metal’s concentration profile, the electron density decreases for the aqueous environment without structures, with only free metals contributing to this value. Because the EDP profiles are also uniformly distributed along the radial direction of the cylindrical structure, we can quantify the number of Ln^3+^ cations per peptide conforming to the macrostructures (χ = [Ln^3+^]/[RTX]), with values given in Figure 4D. This quantification is achieved by dividing the electron density corresponding to the lanthanide component by that of the peptide component, providing a spatially quantitative measurement of metal incorporation within the aggregates. The χ values from solutions containing excess Tb^3+^ or Lu^3+^ showed that within the self-assembly structures, the peptide coordinates with 8.1 ± 0.5 Tb^3+^ or 7.7 ± 0.3 Lu^3+^. These values of χ are in close agreement with the coordination state of the Ln^3+^-peptide in solution^41^ and the crystallographic structures of the Ca^2+^-peptide complexes.^66^ These results suggest that the Ln^3+^-bound peptide is stable within the macrostructures and aggregated cation-peptide complexes are in a similar Ln-coordinated state to the bound state of monomers. Moreover, the number of trivalent cations, χ, coordinating with one RTX domain peptide is sufficient to increase the net charge of the unbound molecule from −23 (based on the charge of the amino acids constituting the molecule at a pH of 5.6) to approximately 0, and in agreement with the zeta potential measurements presented in Figure 2C.

Although the separation of REEs from non-REEs is of significant importance, separating these metals from each other is crucial due to their distinct applications that are vital for different applications. ASAXS measurements of aggregated structures from a solution containing 20 μM of RTX peptide and a mixture of Tb^3+^ and Lu^3+^ at equimolar concentrations (total Ln^3+^ concentration of 500 μM) determine the macrostructures of the peptides coordinated with Tb^3+^ and Lu^3+^ cations with similar affinities to those observed in solution (see Figure S10 for ASAXS profiles and fits). Although the association constant of the peptide for Tb^3+^ and Lu^3+^ are similar, and larger differences in *K*_*D*_ are observed between heavy and light metals,^41^ we used two heavy metals to study selective binding in macrostructures because the absorption edge of light lanthanides lies over the energy range for scattering measurements. Results obtained from fitting the ASAXS scattering components of the solution containing the mixture of Tb^3+^ and Lu^3+^ detailed above, indicate the presence of aggregates with morphology, metal distribution, and electron density along the macrostructures, similar to those of the simple components (see Figure S11 and Table S1). The radial distributions of the aggregates are provided in Figure S12. Values of χ_Lu_ = 3.6 ± 0.2 and χ_Tb_ = 4.1 ± 0.1 were also calculated from fitting the ASAXS terms. The calculated Lu^3+^ to Tb^3+^ ratio of 1.14 ± 0.07, obtained from the χ values derived from the ASAXS analysis, is in agreement with the Tb-bound peptide:Lu-bound peptide ratio of 1.13, obtained from apparent dissociation constants reported for this peptide.^41^ These results suggest that the assembly of individual metal-peptide complexes into the observed micrometer-sized structures does not induce a significant change in the metal-coordination and conformation of the bound peptide. The separation of metals from an equimolar mixture of Tb^3+^, Ca^2+^, and Co^2+^ (1 mM each) with increasing concentrations of RTX was also studied using ICP-OES to simulate a more complex separation scenario. Calcium was selected because it is the native binding partner of the peptide, while cobalt was included as a representative transition metal commonly found in significant quantities in electronic waste, a relevant feedstock for lanthanide recovery. Figure 5 shows a strong enrichment of terbium in the recovered material relative to that of calcium and cobalt, demonstrating the potential of our system to selectively recover lanthanides even in the presence of competing metal ions. Although a wider study on the lanthanide coordination stability of these biomolecules upon macrophase separation is necessary, the results presented here provide the scientific basis for how RTX peptides, upon complexation with lanthanides, undergo aggregation and phase separation. This establishes a promising foundation for developing biobased processes for REE separation.

**Figure 5:**
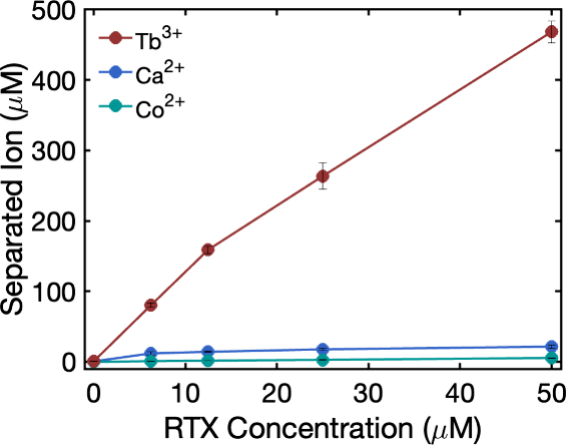
Recovered ion from an equimolar mixture of Tb^3+^, Ca^2+^, and Co^2+^ (1 mM each) as a function of increasing concentrations of RTX at pH 5.6. The concentration of each separated ion was calculated by subtracting the concentration remaining in the supernatant after incubation with the peptide and centrifugation from the initial concentration of the ion in solution. The error bars represent the standard deviations from three independent measurements.

## Conclusions

We showed that the RTX domain can be used for sequestration and biomolecular precipitation of lanthanide cations and could be potentially employed as an alternative separation process. The RTX domain can coordinate with trivalent REE cations and form a folded and stable structure. In the lanthanide-bound state, reduced screening forces promote the formation of self-assembling structures enriched with Ln^3+^ cations. These dense aggregates form spontaneously in solution as the concentration of trivalent ions increases, accumulating at the bottom of the system without the need for centrifugation. While conformational changes of the RTX domain upon metal binding have been established in previous studies, our work provides new insight into how charge screening by lanthanides can drive mesoscale aggregation and condensation. These findings offer a foundation for engineering biomolecules for applications such as reversible and controllable metal separation. Future studies will focus on the separation of lanthanide cations from complex mixtures that mimic industrial feedstock leachates and recycled materials.

## Experimental Section

### Chemicals

All chemicals were purchased from Sigma-Aldrich (St. Louis, MO) unless otherwise specified.

### Protein Expression and Purification

Protocols and methods were adopted mainly from Szilvay et al.^39^ and Bulutoglu et al.^67^ The RTX domain was expressed by using the pMAL expression vector. The peptides were fused to the maltose-binding protein (MBP) and a self-cleaving intein domain and were expressed in 5-alpha cells (New England Biolabs, Ipswich, MA). Cells were cultured in sterilized Terrific Broth supplemented with 100 μg/mL ampicillin and 2 g/L glucose. The cells were grown to the mid-log growth phase and then induced with 0.3 mM IPTG for 4–5 h in a biological shaker at 37 °C. Cells were harvested by centrifugation at 5,000 rpm for 15 min. The cell pellets were resuspended in MBP column buffer (20 mM Tris-HCl, 200 mM NaCl, 1 mM EDTA, pH 7.4) and lysed via sonication. The cell lysate was centrifuged at 10,000 g for 1 h at 4 °C, and the target proteins in the soluble fraction were loaded onto an amylose resin chromatography column (MBPTrap, Cytiva). The column was washed with 20 column volumes using the MBP column buffer, and the MBP fusions were eluted using MBP Elution buffer (20 mM Tris-HCl, 200 mM NaCl, 10 mM Maltose, 1 mM EDTA, pH 7.4). The eluted protein was concentrated to 1 mL using a 50 kDa Amicon Ultra centrifugal filter Ultra-15 (Millipore Sigma, Burlington, MA), diluted to 50 mL with Intein-cleaving buffer (150 mM NaCl, 10 mM KH_2_PO_4_, 40 mM bis-Tris, 2 mM EDTA, pH 6), and incubated on a shaking plate at 37 °C for 16–20 h. The cleaved proteins were concentrated using a 10 kDa MWCO Amicon ultra centrifugal filter and separated from the maltose-binding protein by size exclusion chromatography using a HiLoad 16/600 Superdex 75 pg column. The proteins were buffer exchanged into assay buffer (20 mM Glycine, 20 mM potassium acetate, and 50 mM potassium chloride, pH 5.6) and stored at −20 °C. The purity of the protein was confirmed via SDS-PAGE, and the average protein yield was approximately 30 mg per liter of culture following purification.

### Dynamic Light Scattering

Dynamic light scattering (DLS) was performed on a Zetasizer Nano ZS instrument (Malvern). One mL of the different solutions was analyzed in plastic cuvettes at 25 °C. The *z*-average diameter and polydispersity index (PDI) were calculated from a cumulants analysis, where the diffusion coefficient of particles is converted into a particle size by using the Stokes–Einstein equation. For each sample, 15 runs were performed across three repeated measurements. The error bars represent the peak standard deviation as calculated by Malvern software. Although three measurements were conducted for each sample, only one representative value is reported, as the mean and standard deviation from all three measurements were consistent and fell within the same range.

### Zeta Potential

Zeta potential measurements were taken using a Zetasizer Nano ZS instrument (Malvern). 700 μL of the different solutions were loaded in folded capillary cells and analyzed at a temperature of 25 °C. Electrophoresis measurements were calculated based on the movement of the particles relative to the liquid where they are suspended in, under the influence of an applied electric field. Zeta potential measurements were then computed by using the Henry equation and the electrophoretic mobility values. For each sample, three independent measurements were conducted under identical conditions. The error bars represent the standard deviations calculated from these three measurements.

### Separation Experiments

The self-aggregation property of the RTX domain was utilized to separate an REE, terbium chloride (TbCl_3_), from major competing components commonly found in mining leachates (calcium chloride, CaCl_2_) and transition metals typically present in electronic waste (represented here by cobalt chloride, CoCl_2_). An equimolar mixture of the three salts (1 mM each) was prepared and incubated with varying concentrations of the RTX domain in the same assay buffer. Following aggregation, the protein pellet was collected by centrifugation at 5,000 × g for 1 min. The supernatant was removed, acidified to 3% nitric acid, and analyzed for ion concentrations via ICP-OES.

### Transmission Electron Microscopy (TEM)

TEM measurements were undertaken on a Tecnai Spirit TWIN TEM electron microscope operated at an accelerating voltage of 120 kV. For bulk structures, 4 μL samples were deposited onto TEM grids (pure carbon on copper mesh, Ted Pella Inc., USA) that were previously treated with a plasma cleaner (Fischione M1070 NanoClean) for 60 s. The sample on the grid was lightly blotted with filter paper and then stained with 2% uranyl acetate solution and blotted once again. The sample was rinsed with water and the excess solution was removed by blotting the edge of the grid with filter paper. At least one sample from each of the following conditions was prepared and imaged: RTX 20 μM, RTX 50 μM, RTX 20 μM + Tb 500 μM, RTX 20 μM + Tb 1000 μM, RTX 20 μM + Tb 3000 μM, RTX 50 μM + Tb 1250 μM, and RTX 50 μM + Tb 3000 μM.

### Cryo-Electron Microscopy (Cryo-EM)

For the cryo-electron microscopy (cryo-EM) experiment, grids were purchased from Ted Pella, Inc. (Prod # 01895-F), which have a lacey carbon support film. All grids were treated for 35 s in Fischione Nanoclean 1070 (70% power) with a mixture of argon (75%) and oxygen (25%). Cryo-EM grids were prepared in Vitrobot Mark IV (Thermo Fisher Scientific) at 21 °C with the following settings: the relative humidity 100%, wait time 5 s, blot time 4 s, blot force 4. Three mL of sample solution was pipetted onto a freshly treated lacey carbon grid. The sample solution was incubated on an EM grid, blotted with filter paper, before being plunged into liquid ethane that was precooled by liquid nitrogen. The cryo-EM grids were then transferred and stored in liquid nitrogen. The cryo-EM grid was transferred in liquid nitrogen into a Gatan 626 cryospecimen holder that was then inserted into the microscope stage. The specimen temperature was maintained at about −170 °C during the data collection. Cryo-EM images were taken with a CETA camera (Thermo Fisher Scientific) and in the low-dose mode of Titan Halo TEM operating at 300 kV (Thermo Fisher Scientific).

### Anomalous Small-Angle X-ray Scattering (ASAXS)

The ASAXS measurements were taken at the 15-ID-D experimental station of NSF’s ChemMatCARS beamline of Advanced Photon Source at Argonne National Laboratory. Condensed aggregates from a solution containing 20 μM of RTX domain peptide and different concentrations of Ln^3+^ cations were loaded in a 0.05 in. diameter, polyimide tube (Cole-Parmer, Vernon Hills, IL). Data frames were collected with 1 s exposure time using a Pilatus 3X 300 K detector with a 1 mm Si chip and a sample-to-detector distance of 3.6 m. ASAXS data were collected at 20 different energies below the X-ray absorption L3 edge of Tb and Lu (7.514 and 9.244 keV, respectively). The scattering patterns were also collected from a solution containing just TbCl_3_ and LuCl_3_ for background subtraction and Glassy Carbon for absolute scale normalization at the same energies as the sample. Different scattering terms (SAXS term, cross term, and resonant term) were obtained from energy-dependent SAXS data following the same process described elsewhere.^68,69^ To identify the distribution of counterions, a Uniform Cylinder model was used for analysis.^64^ The length and the radius were initially based on the dimensions obtained from TEM imaging. Fitting with this model was performed by varying these two dimensions, the density of self-assembling structures, and the density of Ln^3+^ cations. A radial distribution function was used to fit the scattering components since the TEM images show structures with a wide distribution of diameters, and the scattering component curves indicate high polydispersity of species in solution. Errors in the fitted parameters were obtained by mapping the χ^2^ space, which quantifies the squared deviation between the scattering data and the corresponding fit for a given set of parameters. This approach enables the assignment of confidence intervals to the fitted values.

## Supplementary Material



## Notes

Intensity-weighted size distributions and autocorrelation functions from DLS measurements; images of RTX–Tb condensates; TEM and cryo-EM micrographs of aggregates; Gaussian probability radial distributions from ASAXS fittings; concentration and electron density profiles from ASAXS analyses; ASAXS scattering data with corresponding fits; and parameters obtained from ASAXS fitting (PDF)
