# Supplementary material for: Rare Earth Element-Induced Condensation of the Block V of the Repeats-in-Toxin Domain from CyaA from Bordetella pertussis for Separations
Source: Langmuir. 2025 Dec 18;41(51):34282–91. doi: 10.1021/acs.langmuir.5c04148 (PMC12756916; doi:10.1021/acs.langmuir.5c04148)
Supplement: Supplementary file 1 [file la5c04148_si_001.pdf]

Supporting Information

Rare Earth Element Induced Condensation of Block V of the RTX Domain from CyaA from *Bordetella Pertussis* for Separations

Luis E. Ortuno Macias[a], Farid Khoury[b], Mrinal K. Bera[c], Wei Bu[c], Binhua Lin[c], Scott Banta[b], Raymond S. Tu[a]*

[a]Department of Chemical Engineering, The City College of New York, New York, 10031, NY, USA
[b]Department of Chemical Engineering, Columbia University, New York, 10027, NY, USA
[c]NSF's ChemMatCARS, Pritzer School of Molecular Engineering, University of Chicago, Chicago, 60637, IL, USA
*Corresponding author
Email: tu@ccny.cuny.edu

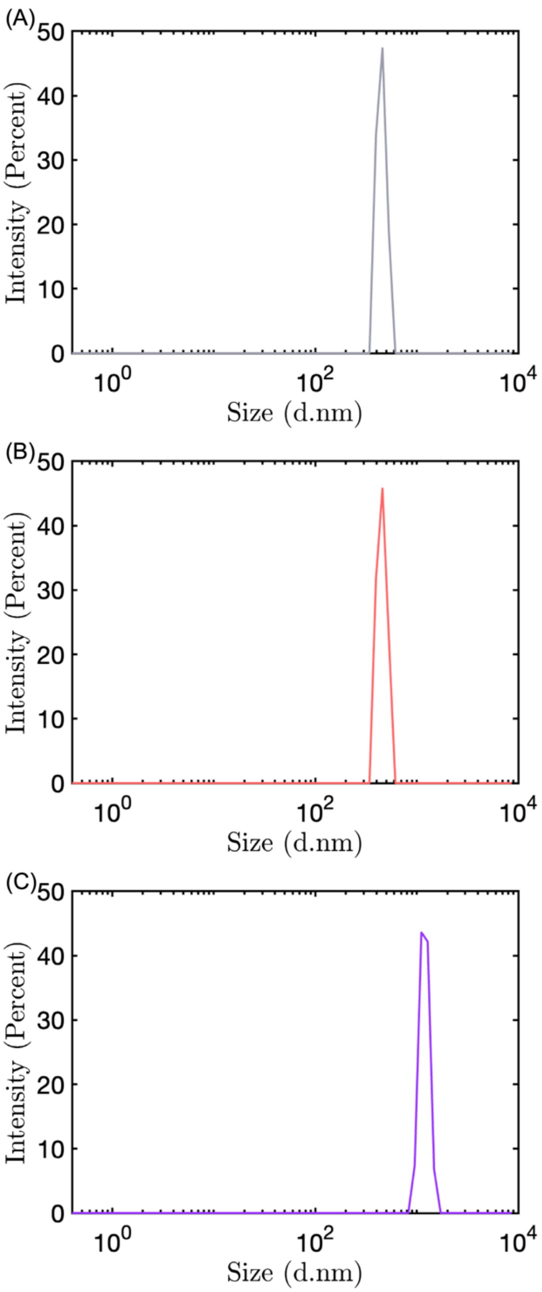

Figure S1. Intensity size distribution for solutions containing (A) 1 μM of RTX domain peptide, (B) 1 μM of RTX domain peptide and 10 mM of Ca^{2+}, and (C) 1 μM of RTX domain peptide and 1 mM of Tb^{3+}.

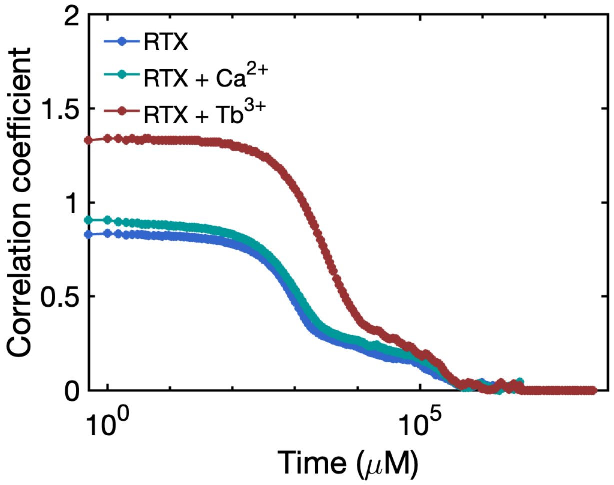

Figure S2. Correlation functions corresponding to DLS from solutions containing 1 μM of RTX domain peptide, 1 μM of RTX domain peptide and 10 mM of Ca^{2+}, and 1 μM of RTX domain peptide and 1 mM of Tb^{3+}.

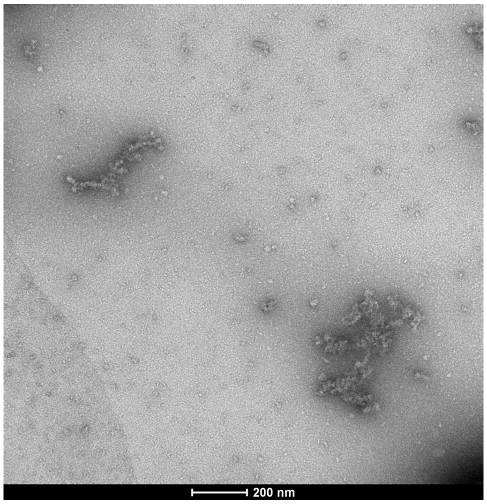

Figure S3. Dry Transmission Electron Microscopy (TEM) structures from solutions containing 20 μM of RTX peptide.

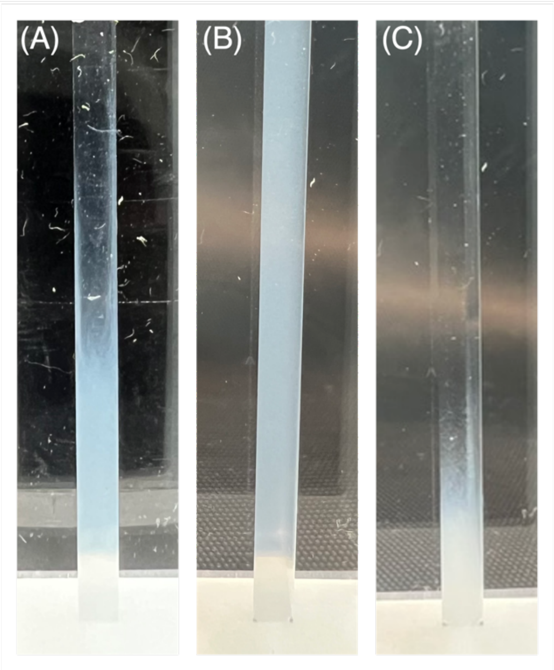

Figure S4. Solutions containing 20 μM of RTX peptide and 0.5 mM of Tb^{3+}, showing the macrophase-separated condensates settled at the bottom of tube. Each panel corresponds to an independent solution.

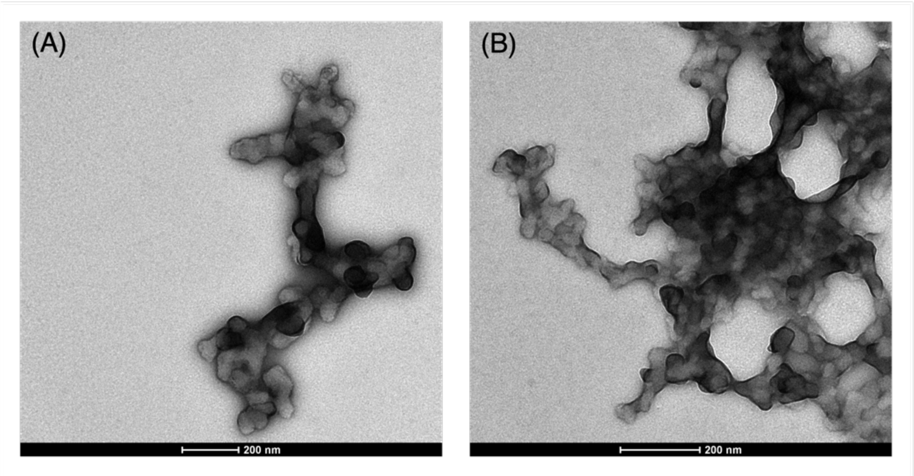

Figure S5. TEM images of dried samples from solutions containing (A) 20 μM of RTX peptide and 1 mM of Tb^{3+}, and (B) 20 μM of RTX peptide and 3 mM of Tb^{3+}.

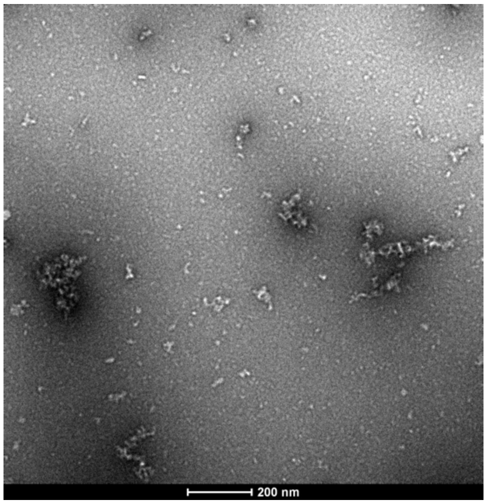

Figure S6. TEM structures from solutions containing 20 μM of RTX peptide and 20 mM of Ca^{2+}.

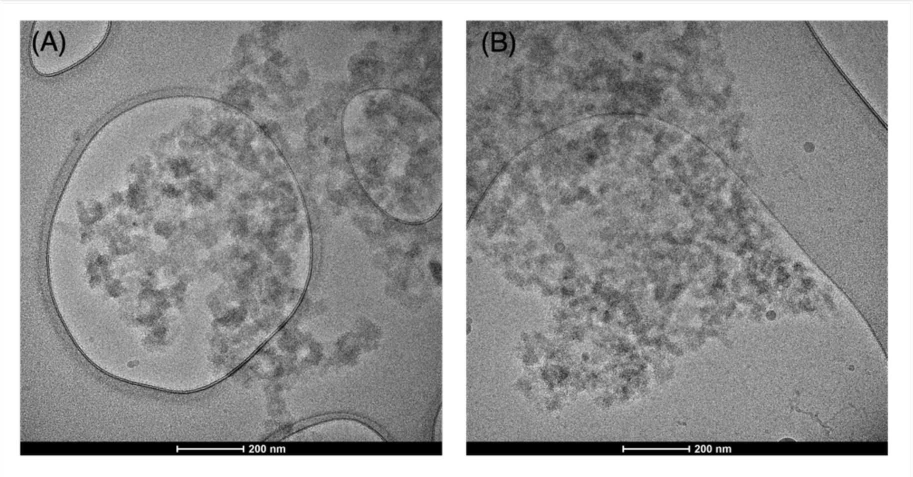

Figure S7. Cryo-electron microscopy (cryo-EM) images of samples from solutions containing (A) 20 μM of RTX peptide and 0.5 mM of Tb^{3+}, and (B) 20 μM of RTX peptide and 0.5 mM of Lu^{3+}.

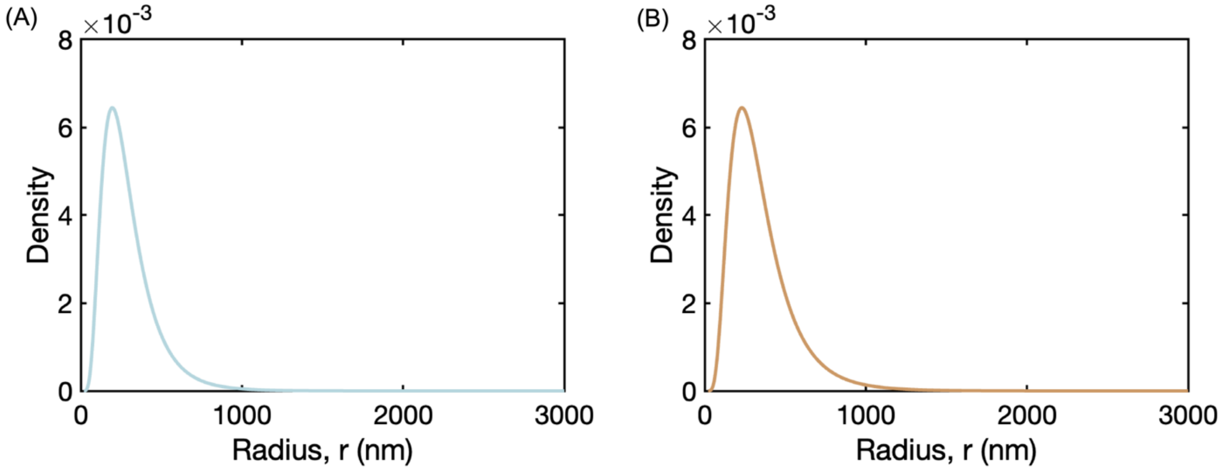

Figure S8. Gaussian probability radial distribution function from solutions containing 20 µM of RTX domain peptide, and 500 µM of Ln^{3+} for (A) Tb^{3+}-RTX aggregates and (B) Lu^{3+}-RTX aggregates.

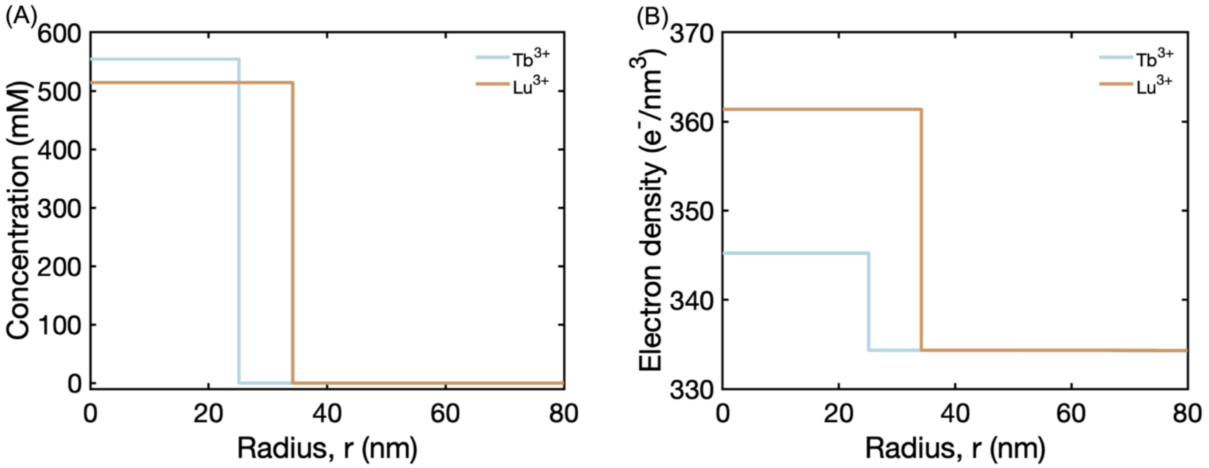

Figure S9. (A) Ln^{3+} concentration profiles from solutions containing 20 µM of RTX domain peptide and either 500 µM of Tb^{3+}, or Lu^{3+}. The lanthanide concentration decreases outside the aggregated structure to 47.8 µM for Tb^{3+} and 14.3 µM for Lu^{3+}, low enough that they appear nearly zero on the plot but reflect the residual presence of ions in the bulk solution. (B) Electron density profiles (EDP) from solutions containing 20 µM of RTX domain peptide and either 500 µM of Tb^{3+}, or Lu^{3+}. Ln^{3+} concentration and EDP are represented as a function of the radial direction of the cylinder, where the lower step function represents the media of the system.

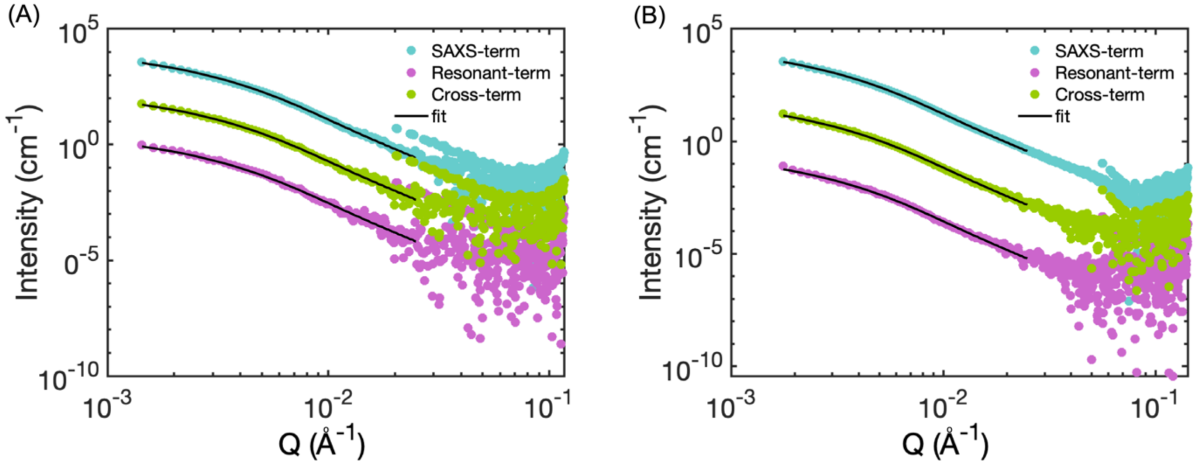

Figure S10. Representative ASAXS profiles and the corresponding fits for a cylindrical model from a solution containing 20 μM of RTX domain peptide, 250 μM of Tb^{3+}, and 250 μM of Lu^{3+}, for (A) Tb^{3+}, and (B) Lu^{3+}.

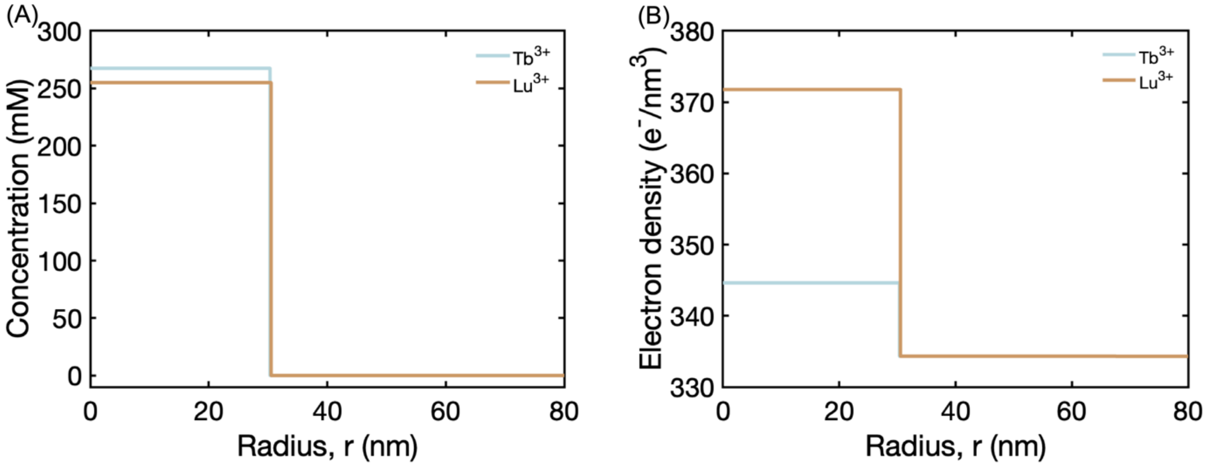

Figure S11. (A) Ln^{3+} concentration profiles from a solution containing 20 μM of RTX domain peptide, 250 μM of Tb^{3+}, and 250 μM of Lu^{3+}. The lanthanide concentration decreases outside the aggregated structure to 55.1 μM for Tb^{3+} and 26.4 μM for Lu^{3+}, low enough that they appear nearly zero on the plot but reflect the residual presence of ions in the bulk solution. (B) Electron density profiles (EDP) from a solution containing 20 μM of RTX domain peptide, 250 μM of Tb^{3+}, and 250 μM of Lu^{3+}. Ln^{3+} concentration and EDP are represented as a function of the radial direction of the cylinder, where the lower step function represents the media of the system.

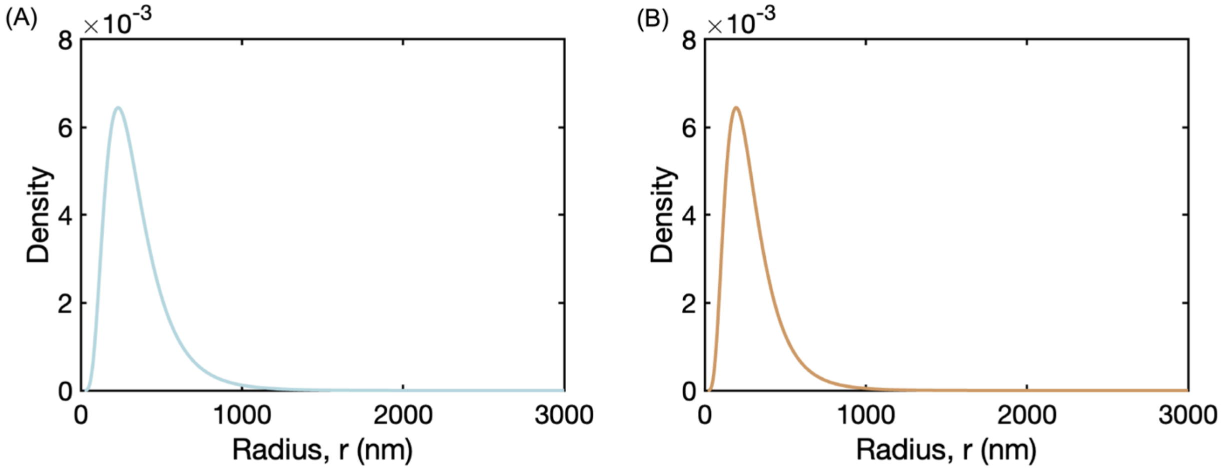

Figure S12. Gaussian probability radial distribution function from a solution containing 20 µM of RTX domain peptide, 250 µM of Tb^{3+}, and 250 µM of Lu^{3+} for (A) Tb^{3+}-RTX aggregates and (B) Lu^{3+}-RTX aggregates.

Table S1. Length and radius of aggregated structures from a solution containing 20 µM of RTX domain peptide, 250 µM of Tb^{3+}, and 250 µM of Lu^{3+}, obtained from ASAXS measurements of the same sample but using different incidents energies (depending on the resonant element of interest). Errors in the fitted parameters are obtained by mapping the chi-squared space.

**RTX peptide = 20 μM**	**Length (nm)**	**Radius (nm)**
**Tb^{3+} = 250 μM**	5136 $\pm$ 35	304 $\pm$ 3
**Lu^{3+} = 250 μM**	4197 $\pm$ 6	305 $\pm$ 1
